# Whole-Genome Draft Sequences of 26 Enterohemorrhagic *Escherichia coli* O157:H7 Strains

**DOI:** 10.1128/genomeA.00134-12

**Published:** 2013-02-28

**Authors:** Mark Eppinger, Sean Daugherty, Sonia Agrawal, Kevin Galens, Naomi Sengamalay, Lisa Sadzewicz, Luke Tallon, Thomas A. Cebula, Mark K. Mammel, Peter Feng, Robert Soderlund, Phillip I. Tarr, Chitrita DebRoy, Edward G. Dudley, Claire M. Fraser, Jacques Ravel

**Affiliations:** a University of Texas at San Antonio, Department of Biology, and South Texas Center for Emerging Infectious Diseases, San Antonio, Texas, USA; b Institute for Genome Sciences (IGS), University of Maryland, School of Medicine, Department of Microbiology and Immunology, Baltimore, Maryland, USA; c Johns Hopkins University, Department of Biology, Baltimore, Maryland, USA; d Center for Food Safety and Applied Nutrition, US Food and Drug Administration, Laurel, Maryland, USA; e National Veterinary Institute, Department of Bacteriology, SVA, Uppsala, Sweden; f Washington University School of Medicine, St. Louis, Missouri, USA; g Penn State University, Department of Veterinary and Biomedical Sciences, University Park, Pennsylvania, USA; h Penn State University, Department of Food Science, University Park, Pennsylvania, USA

## Abstract

First identified in 1982, *Escherichia coli* O157:H7 is the dominant enterohemorrhagic serotype underlying food-borne human infections in North America. Here, we report the genomes of twenty-six strains derived from patients and the bovine reservoir. These resources enable detailed whole-genome comparisons and permit investigations of genotypic and phenotypic plasticity.

## GENOME ANNOUNCEMENT

The Shiga-toxin-producing, non-sorbitol-fermenting, and β-glucuronidase-negative *Escherichia coli* (STEC) O157:H7 strain is thought to have evolved from an O55:H7-like progenitor (1, 2). The O157:H7 serotype is now the most common enterohemorrhagic *E. coli* (EHEC) found in North America. Although it causes disease in humans, *E. coli* O157:H7 asymptomatically colonizes cattle, which is[Table T1]the major reservoir for this organism (3, 4). *E. coli* O157:H7 is distinguished from other serotypes by its genetically homogenous population structure and it exhibits a high degree of proteome conservation and a syntenic chromosomal backbone disrupted by interspersed phages (5, 6). The rapid emergence of *E. coli* O157:H7 from being an unknown strain in 1982 to being the dominant hemorrhagic serotype in the United States and the cause of widespread outbreaks of human food-borne illness highlights a need to critically evaluate the extent to which the genomic plasticity of this important enteric pathogen contributes to human disease severity and bovine niche adaptation. An estimated 15 to 20% of infected patients present with indications severe enough to require hospitalization. Symptoms may progress to hemolytic uremic syndrome, renal failure, hemorrhagic colitis, and central nervous system failure, with potentially lethal outcomes. Yet, little is known about the genomic diversity that exists among extant *E. coli* O157:H7 populations or how various genotypes of this pathogen relate to the development and severity of the clinical manifestations in infected patients.

**TABLE 1 T1:** Genome features of the 26 EHEC strains

Strain	Genome size (Mbp)	Contigs	Accession no.
EC1734	5.42	54	AKMO00000000
EC1738	5.47	62	AKMN00000000
EC4013	5.35	463	AKMH00000000
EC4203	5.39	552	AKMB00000000
EC4402	5.44	785	AKMI00000000
EC4421	5.31	437	AKMF00000000
EC4422	5.31	393	AKMG00000000
EC4436	5.41	456	AKMK00000000
EC4439	5.4	534	AKMJ00000000
EC4448	5.49	566	AKMM00000000
FDA505	5.35	540	AKKW00000000
FDA517	5.52	533	AKKX00000000
FRIK1985	5.54	730	AKKZ00000000
FRIK1990	5.51	629	AKLA00000000
FRIK1996	5.43	580	AKKY00000000
PA3	5.36	508	AKLC00000000
PA5	5.35	554	AKLD00000000
PA9	5.42	427	AKLE00000000
TW07945	5.36	404	AKLU00000000
TW09098	5.48	486	AKLX00000000
TW09109	5.57	66	AKLY00000000
TW09195	5.46	1288	AKLZ00000000
TW10119	5.55	56	AKMA00000000
TW10246	5.45	47	AKLV00000000
TW11039	5.6	71	AKLW00000000
TW14301	5.29	485	AKME00000000

Strains for sequencing were selected to represent phylogenetically diverse isolates within the O157:H7 lineage based on multiple typing assays (1, 5–7). Genomic DNA was subjected to next-generation Illumina HiSeq 2000 (300-bp insert size, 100-bp paired-end reads) or Illumina and 454 FLX/XLR (3-kb insert size) hybrid sequencing followed by assembly as described previously (8). Hybrid and Illumina assemblies were generated using the Celera and Velvet assemblers, respectively (9, 10), and all chromosomes and plasmids were manually annotated using the Manatee system (http://manatee.sourceforge.net/). Genome architectures and gene inventory were compared using Mauve and BLAST Score Ratio analysis (11, 12). Strains were further characterized using phylogenomic assays of the genomic backbone and mobilome to investigate plasticity in architectures, prophage profiles, and single nucleotide polymorphisms (5, 6). Access to these high-quality genome sequences and their comparative analyses with relatives or other serotypes will facilitate additional comprehensive bioinformatics and phylogenetic analyses, thus expanding our understanding of the pathogenomic evolution of this major public health problem caused by these pathogens. These data should also prove useful for the development of a refined phylogenomic framework for forensic, diagnostic, and epidemiological studies in order to better prepare for future outbreaks and for better risk assessment in response to novel and emerging *E. coli* O157:H7 biotypes.

### Nucleotide sequence accession numbers.

The genome sequences are deposited in GenBank under the accession no. listed in Table 1. Cultures are available from the Biodefense and Emerging Infections Research Resources Repository (http://www.beiresources.org/).
